# Bridging the Workforce Gap in Ethiopian Catheterization Laboratories

**DOI:** 10.1016/j.jscai.2025.103803

**Published:** 2025-08-14

**Authors:** Mikiyas G. Teferi, Elias A. Tesfaye, Abenezer A. Wolde, Nahom D. Gerer, Abdi D. Gashu, Abigael A. Mesfin

**Affiliations:** School of Medicine, College of Health Sciences, Addis Ababa University, Addis Ababa, Ethiopia

**Keywords:** clinical care, education or training, research, Ethiopia, interventional cardiology, policy

## Introduction

Cardiovascular disease (CVD) burden is becoming a big problem in sub-Saharan Africa and Ethiopia, yet services from interventional cardiology are not widely accessible. Africa has only about 2000 cardiologists for its 1.2 billion people, there is a big lack of specialists, particularly in advanced areas such as interventional cardiology.^1^ In such countries, the number of interventional cardiologists is in single digits, and catheterization laboratories are also hard to find because they are required at a ratio of 1 for every million people.^2^^,^^3^ Ethiopia demonstrates this problem well: even with more than 130 million people, until recently, only a few centers in the capital offered interventional cardiology. The lack of registered nurses in acute care units results in delayed treatment and unmet treatment needs for patients with ST-elevation myocardial infarction, where the percutaneous coronary intervention needed is often unavailable in urgently needed cases. Addressing this gap is crucial for improving cardiovascular outcomes. This viewpoint highlights problems in Ethiopia’s catheterization laboratory workforce and proposes strategic solutions through training initiatives and technology integration.

## Workforce challenges in Ethiopian catheterization laboratories

Ethiopia is experiencing a serious scarcity of interventional cardiologists and related professionals. There are only 5 cardiothoracic surgeons to serve over 130 million people,^3^ and the number of practicing interventional cardiologists is similarly low. Most advanced heart care is offered only at hospitals in Addis Ababa, so people living in rural Ethiopia have nowhere nearby to receive these services. As a result of these risks, most patients with coronary artery disease do not receive quick interventions. In recent cases of acute coronary syndrome at 2 Addis Ababa hospitals, percutaneous coronary intervention was done in less than 12%.^4^ Ethiopia has usually depended on teams from other nations for medical aid, and patients were also taken abroad when they needed complex heart care.^3^ These short-term fixes have saved many lives, but they prove there is not a strong local workforce. Because Ethiopia lacked specialized training in cardiology, doctors went abroad to study, so there were generally not enough specialists around and occasional losses due to brain drain. Catheterization laboratory nursing and technician positions are hard to fill because of shortages. Collectively, these factors result in catheterization laboratories functioning only part-time and long waiting lists for cardiac procedures.

## Strategic training and capacity building

Growing the number of skilled workers in interventional cardiology is considered very important. Since Addis Ababa University hosts the main medical school and teaching hospital in the country, it is poised to lead in creating local training courses. Number of specialists can be increased by establishing interventional cardiology fellowship in Ethiopia in collaboration with international partner institutions. Models that have been proven in other African countries support this method. For example, the Uganda Heart Institute catheterization laboratory was dependent on visiting cardiologists. After a well-designed training process, the cases were being managed by their own team of Ugandan specialists just 8 years later.^5^ Nurses and general practitioners can be trained to handle certain diagnostic or postprocedural tasks under cardiologist supervision.^1^ Other African regions have found benefit from short-term intensive courses and workshops such as pacemaker training and interpreting imaging. Long-term twinning partnerships help build capacity in advanced procedures and ensure knowledge transfer. An excellent example is Aswan Heart Centre in Egypt, where cardiologists from the diaspora community set up a top cardiac center by regularly training local teams and, over time, allowing them to run the center themselves. Ethiopia could achieve faster training by engaging Ethiopian diaspora cardiologists and by creating partnerships with organizations like the Pan-African Society of Cardiology.^1^ In addition, developing specialized curricula and simulation laboratories for catheterization laboratory nurses and technologists will create the support system needed to expand catheterization laboratory services. Lastly, ensuring these trained personnel remain in their country is equally important. This can be achieved by offering them incentives and career development opportunities.^1^^,^^6^

## Technology integration to augment care

**Figure 1:**
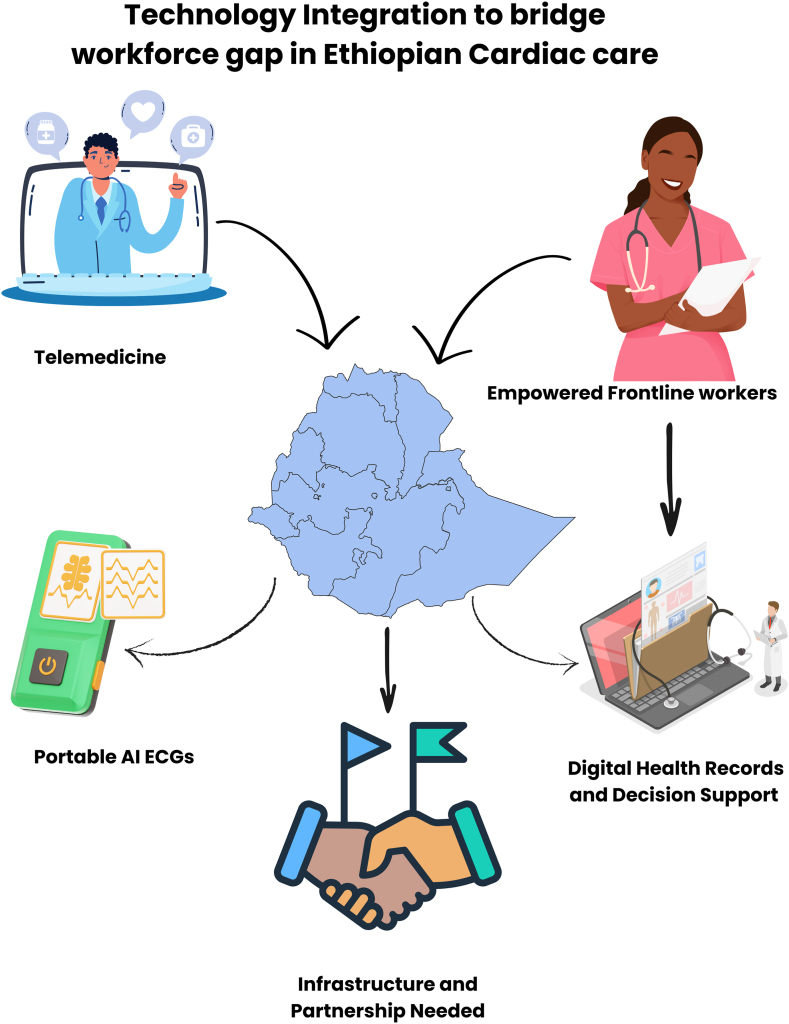
**Technology integration to bridge the workforce gap in Ethiopian cardiac care.** AI, artificial intelligence; ECG, electrocardiogram.

Telemedicine and artificial intelligence (AI) can immediately help close the gap in health care workers. Because there are not many specialist doctors in Ethiopia, telecardiology helps these doctors support distant hospitals. Doctors in the country’s regional centers can address urgent cases with help from guidance from cardiologists in Addis Ababa (Figure 1). AI is becoming an important tool for diagnosis in places with fewer resources. Using AI, portable devices for electrocardiograms can reliably identify arrhythmias and acute ischemia in African communities.^6^^,^^7^ In a pilot carried out in semirural Ethiopia, atrial fibrillation cases went unnoticed when a single-lead smartphone electrocardiogram and AI algorithm were not used. Similarly, using AI helped nonexpert workers detect rheumatic heart disease with great accuracy, as was demonstrated in an East African study.^7^ Using such tools, Ethiopia’s health system is able to allow nurses, health officers, and community health workers to check the heart, sort patients by urgency, and initiate treatment, which lowers the amount of care required from scarce cardiologists.^8^

With the use of patient data systems and assistance applications based in the cloud, work in catheterization laboratories can be performed faster and better.^9^ These innovations make it possible to better choose patients and arrange procedures when needed, so there is maximum use of limited resources.^9^^,^^10^ Even so, before technology can greatly help, clear basic investments are necessary: a dependable electricity supply, up-to-date internet, and proper data management. Cooperation with the Ministry of Health and overseas financiers can allow parallel investment in information technology and training for medical staff. In general, technology does not take the place of experts, but it is an essential addition. Using telemedicine and AI technology in cardiovascular care allows Ethiopia to provide more effects for each cardiologist and nurse and reduce the distance from essential care to underserved populations.^10^

## Conclusion

Strategic workforce training and technology integration are essential to expand equitable access to interventional cardiology and improve cardiac care outcomes across Ethiopia.
